# Prediction of coreceptor usage by five bioinformatics tools in a large Ethiopian HIV-1 subtype C cohort

**DOI:** 10.1371/journal.pone.0182384

**Published:** 2017-08-25

**Authors:** Amare Worku Kalu, Nigus Fikrie Telele, Solomon Gebreselasie, Daniel Fekade, Samir Abdurahman, Gaetano Marrone, Anders Sönnerborg

**Affiliations:** 1 Division of Clinical Microbiology, Department of Laboratory Medicine, Karolinska Institute, Stockholm, Sweden; 2 Department of Microbiology, Immunology and Parasitology, Addis Ababa University, Addis Ababa, Ethiopia; 3 Department of Internal Medicine, Addis Ababa University, Addis Ababa, Ethiopia; 4 Public Health Agency of Sweden, Solna, Sweden; 5 Unit of Infectious Diseases, Department of Medicine Huddinge, Karolinska Institutet, Stockholm, Sweden; University Hospital Tuebingen, GERMANY

## Abstract

**Background:**

Genotypic tropism testing (GTT) has been developed largely on HIV-1 subtype B. Although a few reports have analysed the utility of GTT in other subtypes, more studies using HIV-1 subtype C (HIV-1C) are needed, considering the huge contribution of HIV-1C to the global epidemic.

**Methods:**

Plasma was obtained from 420 treatment-naïve HIV-1C infected Ethiopians recruited 2009–2011. The V3 region was sequenced and the coreceptor usage was predicted by five tools: Geno2Pheno clinical–and clonal–models, PhenoSeq-C, C-PSSM and Raymond’s algorithm. The impact of baseline tropism on antiretroviral treatment (ART) outcome was evaluated.

**Results:**

Of 352 patients with successful baseline V3 sequences, the proportion of predicted R5 virus varied between the methods by 12.5% (78.1%-90.6%). However, only 58.2% of the predictions were concordant and only 1.7% were predicted to be X4-tropic across the five methods. Compared pairwise, the highest concordance was between C-PSSM and Geno2Pheno clonal (86.4%). In bivariate intention to treat (ITT) analysis, R5 infected patients achieved treatment success more frequently than X4 infected at month six as predicted by Geno2Pheno clinical (77.8% vs 58.7%, P = 0.004) and at month 12 by C-PSSM (61.9% vs 46.6%, P = 0.038). However, in the multivariable analysis adjusted for age, gender, baseline CD4 and viral load, only tropism as predicted by C-PSSM showed an impact on month 12 (P = 0.04, OR 2.47, 95% CI 1.06–5.79).

**Conclusion:**

Each of the bioinformatics models predicted R5 tropism with comparable frequency but there was a large discordance between the methods. Baseline tropism had an impact on outcome of first line ART at month 12 in multivariable ITT analysis but only based on prediction by C-PSSM which thus possibly could be used for predicting outcome of ART in HIV-1C infected Ethiopians.

## Introduction

Human immunodeficiency virus type 1 (HIV-1) enters cells through the use of the CD4 receptor and one of the two coreceptors, CCR5 or CXCR4 [1, 2]. These variants are termed R5 and X4 virus, respectively [3]. Initially, determination of strain tropism was done by phenotypic assays [4], but several factors such as cost and turn-around time have limited their clinical use. In contrast, genotypic tropism tests (GTT), which allow computational prediction of coreceptor usage by sequencing the V3 region of the envelope of HIV-1 has become wide-spread. Prediction methods include the charge rule (11/24/25) and several machine learning classification algorithms, including position specific scoring matrix (C-PSSM), support vector machine (SVM) based (e.g. Geno2Pheno) and PhenoSeq. Their performance is influenced by the training data used and most are based on HIV-1 subtype B (HIV-1B) [5], posing a question about their prestanda in non-B subtypes.

Studies validating GTT report decreased sensitivity to detect X4 tropic viruses and differences between subtypes when compared to phenotypic assays [6]. We recently showed that the HIV-1 subtype C (HIV-1C) epidemic in Ethiopia is monophylogenetic and found a discordance between the predicted tropism based on the clonal and the clinical models of Geno2Pheno [7]. Moreover, Ethiopian HIV-1C strains exhibit several unique characteristics in other parts of the genome which are likely to influence their replication characteristics [8, 9].

The increasing influx of migrants to European countries has resulted in an increase of compartmentalized epidemics of non-B subtypes and recombinants forms [10], especially HIV-1C, in several countries e.g. Sweden [11], United Kingdom[12] and Greece[10]. In addition, intracountry cross-transmission between heterosexually infected and men who have sex with men contributes to the increased number of circulating HIV-1C strains [10, 13]. Evaluations of the utility of different bioinformatics for GTT in HIV-1C are thus warranted. Also, R5 and X4 viruses exhibit distinct biological properties, e.g. with regard the replication kinetics, and a preferential selection of X4 virus has been reported in HIV-1B patients on antiretroviral therapy (ART)[14]. However, no study has evaluated the impact of the viral co-receptor tropism on the outcome of non-maraviroc containing ART in low-middle income countries. We therefore investigated the degree of concordance between different bioinformatics methods in a large Ethiopian HIV-1C infected cohort and correlated the findings to outcome of antiretroviral therapy (ART).

## Material and methods

### Study sample

A cross-sectional study was conducted on a random subsample of ART naïve adults (age >16 years) (n = 420) enrolled in the large countrywide Ethiopian Advanced Clinical Monitoring of HIV (ACM) cohort, recruited between 2009 and 2011[7, 15]. The study sample was selected from six geographically distinct clinic sites affiliated to medical universities in Ethiopia (Harar, Eastern Ethiopia; Jimma, Western Ethiopia; Gondar and Mekele, Northern Ethiopia; Hawassa, Southern Ethiopia; Addis Ababa, Central Ethiopia), and a clinic treating the Ethiopian military, the Mobile Group. Subjects were selected randomly, stratified by site, 60 participants from each site. An additional seven non-randomized patients were included only for the study of coreceptor switch at ART failure. The first line ART used consisted of 2 nucleoside analogue reverse transcriptase inhibitors (stavudine or zidovudine or tenofovir and 3TC) and one NNRTI (efavirenz or nevirapin).

The plasma samples were temporarily stored at -20°C at each site, transported to the central laboratory of the Ethiopian Health and Nutrition research institute (EHNRI) and stored at -80°C. HIV-1 RNA load (VL) was analysed by MT 2000 real time PCR (Abbott, USA) and CD4+ T-cell count was enumerated using FACSCount (Becton Dickenson), according to standard operating procedures at EHNRI.

Ethical approval for the study was obtained from the Ethiopian Ministry of Science and Technology and the EHNRI institutional review board. Written informed consent was obtained from all participants.

### Genotypic coreceptor tropism testing

HIV-1 RNA was extracted from plasma using QIAamp Viral RNA kit (Qiagen, Hilden, Germany), according to the manufacturers' instructions. cDNA was synthesized using RevertAid H-minus reagents (Life technologies, Paisley, UK) following the manufacturer’s instructions (Life technologies). The *env* gene encoding the V3-V4 region was amplified from cDNA based on a nested PCR protocol as described previously [16]and with modified primers adapted for HIV-1C_Et_ (https://mullinslab.microbiol.washington.edu/HMA/HMA_Manual.pdf). The first-round PCR was performed with primer ED5 (forward) 5’-ATGGGATCAAAGCCTAAAGC CATGTG-3’ (HXB2 nt 6556–6581) and ED12 (reverse) 5’AGTGCTTCCTGCTGCTCCCAAGA-ACCCAAG-3’ (HXB2 nt 7822–7792) primers to amplify 1200 bp, and inner primers were Et_ES7 (forward) 5' TTRTTAAATGGTAGTATAGC-3' (HXB2 nt 7001–7020) and ES8 (reverse) 5’-CACTTCTCCAATTGTCCCTCA-3’ (HXB2 nt7647-7667) to amplify 650 bp. Second round PCR amplicons were purified using QIAquick kit (Qiagen) followed by bidirectional population sequencing in automated sequencer (ABI 3130xl Genetic Analyzer, Applied Biosystems). Sequences results were aligned, edited, and analyzed using the BioEdit software v.7.0.9.

Prediction of coreceptor usage was performed using five bioinformatics tools: Geno2Pheno clinical–and clonal–models [17], PhenoSeq-C [18], C-PSSM [19], and Raymonds algorithm[20]. Geno2Pheno is optimized for HIV-1B, but a recent study showed a 95% specificity for predicting X4-tropism in HIV-1C obtained from patients in India [21]. PhenoSeq-C, and C-PSSM are optimized for HIV-1C. Raymond’s algorithm is genotypic rule combining the amino acids at positions 11/25 and the net charge of V3 tested for HIV-1C.

As bulk sequencing often produces sequences with sites of ambiguity whereby two or more nucleotides occur with similar frequency, V3-nucleotide sequences were used as input for PhenoSeq-C, which along with the tropism prediction converts bulk nucleotide V3 sequences containing ambiguity into multiple unambiguous amino acid sequences, by generating and translating all possible nucleotide combinations [18]. The obtained amino acid sequences were used as an input for the other algorithms. PhenoSeq-C and C-PSSM provide a binary answer: the virus can use X4 or the virus cannot use X4. For Geno2Pheno, a false positive rate (FPR) below 10% was considered as X4-tropic strains and coded in binary value. The virus was considered “X4 tropic” or “R5-tropic” if all sequences from a particular patient predicted to be X4 or R5-tropic, respectively, in the above-mentioned tools. Virus that were predicted to contain sequences from both X4- and R5-tropic viruses were classified as mixed “R5/X4-tropic”.

### Statistical analysis

Baseline socio-demographic and laboratory characteristics (gender, age, year of HIV diagnosis, year of enrolment, CD4+ T-cells, VL) were analysed. The treatment outcome was assessed with both intention-to-treat (ITT) and on-treatment (OT) analysis. In the ITT analysis, ART failure was defined as either detectable VL (>1000 copies/ml), death or lost-to-follow-up (LTFU). In the OT analysis, we include only those who had actual VL data at the follow up time points and excluded patients who were dead or LTFU. Descriptive analyses included frequencies for categorical variables, mean and standard deviation or median and interquartile range for continuous variables. Chi-square test or Fisher’s Exact Test were used to test differences between categorical variables. Independent t-test, Mann-Whitney, Anova and Kruskal-Wallis test assessed differences of numerical variables between two or more categories. Logistic regression models were used for the multivariable analysis of virological responses to compare differences between R5 and X4 infected patients as predicted by 4 different methods, adjusting by age and gender, baseline CD4+ T-cell count and VL. Odds Ratios (OR), 95% Confidence interval and p-values were used to present the regression models results. Results were adjusted for design effect due to the cluster nature of the study design. P-values<0.05 were considered significant. Data analysis was done by the STATA software 13 (Stata Corp. College Station, USA) and IBM SPSS Statistics, version 22 (IBM Corp).

## Results

### V3 loop sequences

At baseline V3 loop sequencing was successful in 352 of 420 (84%) patients. No difference was found in viral load or other characteristics between patients who had a successful sequencing or not. The length of the V3 loop was 35 amino acids (aa) in 333 (94.6%) of the 352 sequences while 15 sequences were 34 aa, two sequences 37 aa long, two sequences 36 and 38 aa each. HIV-1C was found in 350 (99.4%) patients and A1 in two (0.6%) patients.

### R5 prediction at baseline

Patients predicted to be infected with R5 or X4 strains were compared with regard to age, gender, CD4+ T-cell count and VL at baseline. No statistically significant difference was found for any comparison, except for the Geno2Pheno clinical model where the CD4+ T-cells were higher in R5 patients (cells/μl: 132 vs 58; P<0.001) (Table 1).

**Table 1:** Baseline characteristics of 352 HIV-1C infected Ethiopians in relation to predicted tropism by five bioinformatics tools.

	All patients	Geno2phenoclinical 10%			Geno2phenoclonal 10%			PhenoSeq-C			C-PSSM			Raymond		
		R5	X4*	P	R5	X4*	P	R5	X4*	P	R5	X4*	P	R5	X4*	P
Number (%)	352	285 (81.0)	67 (19.0)		279 (79.3)	73 (20.7)		291 (82.7)	61 (17.3)		275 (78.1)	77 (21.9)		319 (90.6)	33 (9.4)	
Mean age±SD	34.9±9.1	34.7±9.1	34.1±9.0	0.585	34.9±9.1	33.5±8.9	0.294	34.7±8.9	34.0±9.6	0.503	34.9±9.1	33.5±8.8	0.294	34.5±9.2	35±8.2	0.498
Median CD4 (IQR)**	120 (62–185)	132 (76–193)	58 (21–109)	<0.001	125 (67–187)	90 (52–160)	0.067	125 (67–189)	91 (44–155)	0.090	119 (64–183)	122 (57–187)	0.706	119 (62–181)	128 (70–210)	0.390
Median VL (IQR)***	5.37 (4.88–5.80)	5.33 (4.82–5.79)	5.43 (5.07–5.83)	0.103	5.37 (4.92–5.84)	5.32 (4.82–5.77)	0.362	5.35 (4.88–5.80)	5.40 (4.82–5.85)	0.398	5.33 (4.90–5.82)	5.28 (4.78–5.77)	0.394	5.37 (4.90–5.81)	5.25 (4.72–5.78)	0.370

*Patients who were predicted to have a mixed R5X4 infection were classified as having X4 virus

** cells/μl

*** log10 copies/ml.

There was a tendency to higher CD4+ T-cells in R5 patients also for the Geno2Pheno clonal model (p = 0.09) and the C-PSSM (p = 0.067), but not for PhenoSeq-C.

The proportion of patients predicted to be infected with R5 virus varied between the methods by 12.5%, the highest (90.6%) by Raymond’s algorithm and the lowest (78.1%) by PhenoSeq-C (Table 1). Also, the proportion of patients predicted to be infected with mixed R5X4 viruses were similar (Geno2Pheno clinical: 2.0%; Geno2Pheno clonal: 3.1%; PhenoSeq-C: 2.3%; C-PSSM: 2.0%). However, when the five methods were compared, only 205 (58.2%) of the predictions were concordant for all five tools (Table 2). The PhenoSeq-C and the Geno2Pheno clinical model showed least concordance (70.5%), while the highest was between C-PSSM and Geno2Pheno clonal model (86.4%).

**Table 2:** Concordance of tropism in HIV-1C infected patients as predicted by five bioinformatics tools, before antiretroviral treatment and at virological treatment failure.

	**Baseline (n = 352)**				**Month 6 (n = 34)**				**Month 12 (n = 19)**			
**Methods**	R5	X4	Total	Concordance	R5	X4	Total	Concordance	R5	X4	Total	Concordance
**All methods**	199	6	205	58.2%	22	2	24	70.6%	14		14	73.7%
**PhenoSeq-C/C-PSSM**	245	40	285	81.0%	24	6	30	88.2%	14	1	15	78.9%
**PhenoSeq-C/G2P clonal**	246	27	273	77.6%	27	5	32	94.1%	15	1	16	84.2%
PhenoSeq-C/G2P clinical*	230	18	248	70.5%								
**C-PSSM/G2P clonal**	263	41	304	86.4%	25	5	30	88.2%	17	1	18	94.7%
C-PSSM/G2P clinical*	236	22	258	73.3%								
G2P clonal/G2P clinical*	245	19	264	75.0%								
**PhenoSeq-C/Raymond**	271	28	299	84.9%	25	2	27	79.4%	15	1	16	84.2%
**C-PSSM/Raymond**	260	14	274	77.8%	23	2	25	73.5%	17	0	17	89.5%
**G2P clinical/Raymond**	269	16	285	81%								
**G2P clonal/Raymond**	273	15	288	81.8%	27	2	29	85.3%	17	1	18	94.7%

*Geno2Pheno (G2P) clinical method was used only in treatment-naïve patients.

### R5 prediction at virological treatment failure

The viral tropism of the patients who failed virologically at month six and 12, respectively, were predicted. At month six (n = 34), the overall concordance was 24/34 (70.6%) with PhenoSeq-C and Geno2Pheno clonal model showing the highest concordance (94.1%) (Table 2). For viral failures at month 12 (n = 19), the concordance was 14/19 (73.7%) with C-PSSM and Geno2Pheno clonal model showing the highest concordance (94.7%) (Table 2).

### Impact of baseline tropism on ART outcome

Of the 352 patients, 33 patients had died, 28 were LTFU and 15 had a missing VL, at month six. Of the remaining 276 (78.4%) patients, 37 (12.7%) had >1000 copies/ml (mean VL log10, copies/ml: 5.06; range: 3.1–7.0). By OT bivariate analysis, there was no significant difference in achieving viral suppression at month six between patients with R5 or X4 virus at baseline, by any method (Table 3).

**Table 3:** Bivariate on-treatment analysis of treatment outcome of patients at month 6 and 12 as tropism predicted by five methods.

**Method**	% success* **month 6 (n = 276)**			**% success month 12 (n = 198)**		
	R5	X4	p-value	R5	X4	p-value
**G2P clin10%**	87.5	84.1	0.624	88.5	87.9	1.000
**G2P clon10%**	86.9	87.2	1.000	89.2	83.9	0.370
**PhenoSeq-C**	86.8	87.5	1.000	89.9	89.9	0.308
**C-PSSM**	87.9	83.0	0.365	90.2	79.4	**0.080**
**Raymond**	90.8	91.7	1.000	90.9	91.3	1.000

* Success defined as achieving viral load of <1000 copies/ml.

Among the 33 patients who were reported to have died at month 6, the proportion of X4 infected patients was 42.4%, 27.3%, 15.2%, 33.3% and 15.2% by G2P clinical, G2P clonal, PhenoSeq-C, C-PSSM and Raymond respectively. Among the 28 LFTU patients he corresponding figures were 14.3% for G2P clinical, G2P clonal and PhenoSeq-C, 17.9% for C-PSSM and 3.6% for Raymond. When patients were grouped as alive, dead or LTFU at month 6, a statistically significant difference (p = 0.001) was found between the groups only when tropism was predicted by G2P clinical.

At month 12, a further 10 patients had died, 28 were LTFU and 55 patients had a missing VL (mean VL: 4.9; range: 3.4–6.8). Of the remaining 198 (56.3%) patients, 22 patients (8.6%) had > 1000 copies/ml and 55 (21.7%) had missing VL. Thus, 176 out of 352 (50.0%) reached treatment success in an ITT–analysis at month 12. By OT bivariate analysis, X4 infected patients, predicted by C-PSSM, achieved viral suppression less frequently although the difference was not statistically significant (90.2% vs 79.4%, P = 0.08). However, no difference was shown between R5 and X4 infected patients at months six and 12 in multivariable OT analysis (Table 3).

In bivariate ITT analysis, a significant difference in treatment outcome between R5 and X4 infected patients was observed at month six by the Geno2Pheno clinical model (77.8% vs 58.7%,P = 0.004) and at month 12 by C-PSSM (61.9% vs 46.6%, P = 0.038) (Table 4).

**Table 4:** Bivariate intention to treat analysis of treatment outcome of patients at month 6 and month 12 as baseline tropism predicted by five methods.

**Method**	% success at month 6 ITT*(n = 324)			**% success at Month 12 ITT(n = 296)**		
	R5	X4	p-value	R5	X4	p-value
**G2P clin10%**	77.8	58.7	**0.004**	60.8	50.9	0.186
**G2P clon10%**	74.5	71.9	0.739	59.8	54.2	0.522
**PhenoSeq-C**	73.3	76.7	0.739	57.1	65.6	0.252
**C-PSSM**	76.6	64.7	0.061	61.9	46.6	**0.038**
**Raymond**	74.7	68.8	0.524	58.9	59.3	0.97

*Intention to treat analysis where treatment failure was defined as viral load >1000 copies/ml, death and LTFU.

The Geno2Pheno clinical tool finding did not remain significant in the multivariable analysis, when adjusted by age, gender, baseline CD4 and VL. Instead, tropism as predicted by C-PSSM had an impact on month 12 in ITT by the multivariable analysis, with patients with R5 tropism 2.47 times more likely to achieve VL suppression compared to patients with X4 tropism (P = 0.04, OR 2.47, 95% CI 1.05–5.79) (Table 5).

**Table 5:** Multivariable analysis of the impact of baseline viral tropism on antiretroviral treatment response by five bioinformatic methods in an intention to treat analysis.

**Follow-up**	**Predictor at baseline**	OR* **Adjusted by age and gender**	**p-value**	OR* **Adjusted by age, gender, baseline CD4 and VL**	**p-value**
**Month 6 n = 324**	Viral tropism by G2P clinical model	2.36 (1.11–5.04)	**0.032**	1.66 (0.90–3.05)	0.091
	Viral tropism by G2P clonal model	1.09 (0.35–3.41)	0.858	0.93 (0.31–2.81)	0.878
	Viral tropism by PhenoSeq	0.79 (0.33–1.87)	0.533	0.77 (0.34–1.77)	0.477
	Viral tropism by C-PSSM	1.70 (0.77–3.75)	0.149	1.61 (0.62–4.17)	0.265
	Viral tropism by Raymond	1.37 (0.62–3.04)	0.434	1.54 (0.66–3.04)	0.316
**Month 12** **n = 296**	Viral tropism by G2P clinical model	1.60 (0.61–4.18)	0.278	1.32 (0.59–2.98)	0.428
	Viral tropism by G2P clonal model	1.80 (0.81–4.01)	0.121	1.70 (0.76–3.79)	0.157
	Viral tropism by PhenoSeq	0.90 (0.56–1.46)	0.612	0.90 (0.53–1.52)	0.640
	Viral tropism by C-PSSM	2.52 (1.12–5.68)	**0.032**	2.47 (1.06–5.79)	**0.040**
	Viral tropism by Raymond	0.99 (0.44–2.21)	0.982	1.05 (0.46–2.42)	0.908

* R5 vs X4 (reference).

Geno2Pheno: G2P.

### Tropism switch at months six and 12

At month six, virological failure occurred in 37 randomized patients and in seven non-randomized additional patients who were included only for the study of coreceptor switch. Plasma samples at baseline and at failure were available in 41 of them. At baseline, V3 sequencing was successful in 34 patients. The most frequent R5 tropic virus prediction was by Raymond’s algorithm (30/34, 88.2%) and the lowest rate by C-PSSM (26/34, 76.5%). The most frequent rate of tropism switch (7/34; 20.6%) was described by C-PSSM (R5 to X4: 4/26; X4 to R5: 3/8; P = 0.017). For the PhenoSeq and Geno2Pheno clonal tools, 4/34 strains switched each (two R5 to X4 and two X4 to R5). For Raymond’s algorithm, 4/34 strain switched (R5 to X4: 3/30; X4 to R5: 1/4)

At month 12, 22 subjects failed virologically in whom V3 sequencing was successful in 19. Of these, nine had also a failure at month six. In most patients, the same tropism was found at month 12 as at baseline (Geno2Pheno clonal model: 16/19; PhenoSeq: 14/19).

## Discussion

HIV-1C contributes to more than 50% of the global epidemic and is the dominating non-B subtype among newly diagnosed in several European countries, e.g Sweden [11], Iceland [22], United Kingdom[12], and Belgium/Luxemburg [23] An important feature of HIV-1C is the very frequent usage of the CCR5 coreceptor, even in the later stage of disease [24–26]. In order to compare the clinical usefulness of five commonly used bioinformatics tools in HIV-1C infected patients, we analysed the V3 loop sequences derived from a large cohort of HIV-1C infected Ethiopians and compared the predicted tropism with clinical data and ART outcomes.

The bioinformatics methods have been developed largely on HIV-1B data, although PhenoSeq-C [18] and C-PSSM [19] have been trained on HIV-1C. In our study, each bioinformatics tool predicted a similar prevalence of R5 viruses in the HIV-1C infected Ethiopians. This is in concordance with previous comparative studies claiming a reliable performance of GTT tools [27–29], with no one clearly performing better than the other [30] when compared to phenotypic assays. Studies comparing the concordance between genotypic tools are however scarce. In our study, large differences were found between the four bioinformatics tools with an overall concordance of 58.2% in 352 treatment naïve patients. This discrepancy is likely due to the use of different statistical models and e.g. how to handle insertions, deletions and ambiguous positions [31]. In agreement with our finding, one study which employed Geno2Pheno (clinical as well as clonal), PSSM, and Raymond’s algorithm reported concordance in 29 of 50 samples (58%) [30]. In contrary, the prediction in patients failing ART seemed to have a better concordance, although only few patients were analysed. A preferential X4-tropic dependent elimination during ART has been reported [14]. Also, we found that a switch from X4 to R5 viruses at failure month six was more common with C-PSSM. If these selection events result in a more homogenous viral population at failure, in connection with a lower viral load, this could possibly explain the higher concordance between the methods.

When comparing the prediction tools pairwise, the C-PSSM and Geno2Pheno clonal models were highly concordant (86.4%), in line with previous studies which demonstrated the best concordance (>85%) between these two tools among several [32–34]. A V3 length other than 35 amino acids have been reported to be the only factor independently associated with prediction disagreement for Geno2Pheno clonal model and PSSM (Seclen et al 2011). However, we could not find any association of any factors including the V3 length with prediction disagreement in our data set. It should be noted that in Seclen’s study nearly 20% of the V3 sequences analysed had amino acid length other than 35 while in our study the vast majority of V3 sequences (94.6%) had amino acid length of 35.

Data regarding impact of baseline tropism on first line, non-maraviroc containing, ART outcome are scarce. Some reports have described poorer viral load suppression or CD4+ T cell increase in patients with X4 virus at baseline [35–38], while others show similar rates [39, 40]. In addition, a recent study showed that presence of CXCR4-using viruses was associated with the virological failure of antiretroviral treatment initiated during primary HIV infection[41]. The clinical value of GTT in HIV-1B has thus not clearly been shown for non-maraviroc containing ART. Also, it has been claimed that tools based on HIV-1B perform well for HIV-1C [20], while others report the contrary [19]. We did not find any significant association of the predicted coreceptor usage to clinical parameters or outcome of ART, for most comparisons. The presence of R5 virus was associated with higher CD4+ T-cells only with the Geno2Pheno clinical model, which also correlated to the month six ART outcome in a bivariate analysis. However, in a multivariate ITT analysis only tropism predicted by C-PSSM correlated to ART outcome, at month 12. Thus, the clinical value for predicting outcome of ART by GTT is limited in an Ethiopian setting. To our best knowledge, this is the first study to analyse the impact of baseline viral tropism on first line ART outcome, using a large data set from a cohort of exclusively HIV-1C infected patients.

There are some shortcomings in our study. We classified patients who were predicted to have a mixed R5X4 infection as having X4 virus in the statistical analysis, despite that no clonal analysis was done, since X4 viruses are uncommonn in HIV-1C patients. However, only 2% of the samples exhibited such a pattern by each prediction method and did not affect the results. We did not compare our genotypic data with a phenotypic method which is usually considered as a gold standard so that determination of sensitivity and specificity of the GTT methods for HIV-1C was not possible. However, the purpose of our study was rather to compare the results with clinical information in the largest cohort of HIV-1C patients analysed so far with GTT. Consequently, we could not identify a single GTT, which correlated best to clinical outcome in HIV-1C patients, although tropism predicted by C-PSSM correlated to outcome at month 12 in an ITT analysis. Moreover, we did not adjust for multiple comparisons since this study focused on only a few scientifically sensible comparisons, rather than every possible comparison. For this reason there is a risk of false positive findings.

## Conclusion

Each of the GTT predicted R5 tropism with comparable high frequency in HIV-1C infected Ethiopians, but there was a large discordance and only 1.7% were X4-tropic by all five methods. Baseline tropism had an impact on outcome of first line ART in only multivariate ITT analysis based on C-PSSM, at month 12, although no difference was found at month 6. Thus, among all of the tropism prediction methods tested, C-PSSM could possibly be used to predict outcome of ART in HIV-1C infected patients.

## Supporting information

S1 File Demographic and clinical information of study subjects at baseline and follow-up points (month 6 and month12). (XLSX)
